# Comparative genomics analysis and virulence-related factors in novel *Aliarcobacter faecis* and *Aliarcobacter lanthieri* species identified as potential opportunistic pathogens

**DOI:** 10.1186/s12864-022-08663-w

**Published:** 2022-06-27

**Authors:** Jiacheng Chuan, Anatoly Belov, Michel Cloutier, Xiang Li, Izhar U. H. Khan, Wen Chen

**Affiliations:** 1 Ottawa Research and Development Centre, Agriculture and Agri-Food Canada, 960 Carling Ave., Ottawa, Ontario K1A 0C6 Canada; 2 Canadian Food Inspection Agency, Charlottetown, PE C1A 5T1 Canada; 3 Department of Biology, University of Prince Edward Island, Charlottetown, PE C1A 4P3 Canada; 4 Department of Biology, University of Ottawa, Ottawa, ON K1N 9A7 Canada

**Keywords:** Antimicrobial resistance, *Aliarcobacter*, *Arcobacter*, Comparative genomics, Toxin, Virulent factors

## Abstract

**Background:**

Emerging pathogenic bacteria are an increasing threat to public health. Two recently described species of the genus *Aliarcobacter, A. faecis* and *A. lanthieri,* isolated from human or livestock feces, are closely related to *Aliarcobacter* zoonotic pathogens (*A. cryaerophilus*, *A. skirrowii*, and *A. butzleri*). In this study, comparative genomics analysis was carried out to examine the virulence-related, including virulence, antibiotic, and toxin (VAT) factors in the reference strains of *A. faecis* and *A. lanthieri* that may enable them to become potentially opportunistic zoonotic pathogens.

**Results:**

Our results showed that the genomes of the reference strains of both species have flagella genes (*fla*A, *fla*B, *flg*G, *flh*A, *flh*B, *fli*I, *fli*P, *mot*A and *che*Y1) as motility and export apparatus, as well as genes encoding the Twin-arginine translocation (Tat) (*tat*A, *tat*B and *tat*C), type II (*pul*E and *pul*F) and III (*fli*F, *fli*N and *ylq*H) secretory pathways, allowing them to secrete proteins into the periplasm and host cells. Invasion and immune evasion genes (*cia*B, *iam*A, *mvi*N, *pld*A, *irg*A and *fur*2) are found in both species, while adherence genes (*cad*F and *cj*1349) are only found in *A. lanthieri*. Acid (*clp*B), heat (*clp*A and *clp*B), osmotic (*mvi*N), and low-iron (*irg*A and *fur*2) stress resistance genes were observed in both species, although urease genes were not found in them. In addition, *arc*B, *gyr*A and *gyr*B were found in both species, mutations of which may mediate the resistance to quaternary ammonium compounds (QACs). Furthermore, 11 VAT genes including six virulence (*cad*F, *cia*B, *irg*A, *mvi*N, *pld*A, and *tly*A), two antibiotic resistance [*tet*(O) and *tet*(W)] and three cytolethal distending toxin (*cdt*A, *cdt*B, and *cdt*C) genes were validated with the PCR assays. *A. lanthieri* tested positive for all 11 VAT genes. By contrast, *A. faecis* showed positive for ten genes except for *cdt*B because no PCR assay for this gene was available for this species.

**Conclusions:**

The identification of the virulence, antibiotic-resistance, and toxin genes in the genomes of *A. faecis* and *A. lanthieri* reference strains through comparative genomics analysis and PCR assays highlighted the potential zoonotic pathogenicity of these two species. However, it is necessary to extend this study to include more clinical and environmental strains to explore inter-species and strain-level genetic variations in virulence-related genes and assess their potential to be opportunistic pathogens for animals and humans.

**Supplementary Information:**

The online version contains supplementary material available at 10.1186/s12864-022-08663-w.

## Background

The genus *Aliarcobacter* (formerly *Arcobacter*) belongs to the family *Campylobacteraceae* in *Epsilonproteobacteria* [1–3]. To date, *Aliarcobacter* consists of nine Gram-negative species reclassified from *Arcobacter* *sensu lato* species, including *A. butzleri*, *A. cibarius*, *A. cryaerophilus*, *A. faecis*, *A. lanthieri*, *A. skirrowii*, *A. thereius*, *A. trophiarum*, and *A. vitoriensis* [2–4]. *Aliarcobacter* species are motile by single polar flagellum and can survive in microaerobic and aerobic conditions [2, 5]. *Aliarcobacter* species have been commonly detected in a variety of foods, including chicken, beef, pork, shellfish, and aquatic niches [6–8], where they can be contaminated by livestock and poultry wastes, agricultural runoff, septic leakages, and wildlife fecal matter [9, 10]. Among the nine *Aliarcobacter* species, *A. butzleri*, *A. cryaerophilus*, and *A. skirrowii* are associated with human and animal infections, including gastroenteritis, bacteremia, sepsis, mastitis, diarrhea, abortion, and reproductive disorders [5]. In addition, antimicrobial susceptibility and the detection of virulence factors confirmed *A. thereius* as a zoonotic pathogen [11–13]. Although the physiology and genetics of *Aliarcobacter* are still poorly understood, comparative genomics analysis can help in deciphering the genetic codes of *Aliarcobacter* species and elucidate their ecological roles and pathogenic potential. It is worth noting that a recent genome-based study proposed to include *Aliarcobacter*, *Halarcobacter*, *Malaciobacter*, *Pseudarcobacter*, *Poseidonibacter,* and *Arcobacter* *sensu stricto* in a single genus, *Arcobacter* [14].

To date, only the genomes of *A. butzleri*, *A. cibarius*, *A. cryaerophilus,* and *A. thereius* have been characterized in detail [11, 15–17]. These genomes are featured as low GC content (ca. 27%), with the genome sizes ranging from 1.8 to 2.3 Mb [11, 15, 17]. Comparative genomics further identified several sets of genes or proteins that may be associated with the pathophysiology of pathogenic *Aliarcobacter* species. Strains of *A. butzleri* often carry a full or partial set of the nine virulence determinants that are homologous to genes with known pathogenic mechanisms, including the putative virulent factor *mviN* [18] or genes associated with adherence (*cad*F, *cj*1349, *hec*A and *irg*A), invasion (*cia*B) or destruction (*hec*B, *tly*A, *pld*A) of host cell walls [5]. Genes or gene clusters involved in the biosynthesis of lipooligosaccharides and flagella, chemotaxis, and antimicrobial resistance have also been identified in *A. butzleri* and *A. thereius* [11, 15]. Genome analysis combined with laboratory experiments suggested that *A. butzleri*, *A. cryaerophilus,* and *A. skirrowii* may survive in cold and oligotrophic environments, disinfection regimes, food process procedures, and storage conditions [5]. It was reported that the antimicrobial resistance of pathogenic *Aliarcobacter* strains might be chromosomally determined and associated with the activity of efflux pumps or the presence of degrading enzymes encoded by genes such as *cat* (chloramphenicol resistance) [5, 11, 12, 15]. It has also shown that mutations in the quinolone-resistance-determining region of *gyr*A mediate bacterial susceptibility to fluoroquinolones [19, 20]. In addition, exotoxins and endotoxins and toxin-antitoxin (TA) systems are critical self-defense mechanisms for bacteria that determine a pathogen’s capacity and persistency of pathogenicity [21].

*Aliarcobacter lanthieri* strain AF1440^T^, AF1430, and AF1581 were isolated from pig and dairy cattle manure [22], and *A. faecis* strain AF1078^T^ was isolated from a human septic tank [23]. *A. lanthieri* and *A. faecis* are phylogenetically closely related and clustered with *A. cryaerophilus*, *A. skirrowii*, and *A. butzleri*, based on the phylogenetic analysis using 16S rRNA and housekeeping (*gyr*B, *rpo*B, *cpn*60, *gyr*A, and *atp*A) genes; and equipped with short flagellum for mobility [22, 23]. Besides, a recent study isolated and identified *A. lanthieri* strain R-75363 from the stool culture of an immunocompetent patient who developed persistent abdominal bloating and cramps without fever or diarrhea [24]. Therefore, the focus of this study was to assess the virulence-related factors of these two species through comparative genomics analysis. The objectives of this study were to i) perform whole-genome assembly of the reference strains of *A. lanthieri* and *A. faecis*; ii) assess the taxonomic position of *A. lanthieri* and *A. faecis* based on genome homology; and iii) identify virulence-, antimicrobial resistance- and toxin-related genes in *A. lanthieri* and *A. faecis.* This study provided information on the antibiotic resistance, virulence potential, and general fitness of these two new *Aliarcobacter* species in natural environments.

## Materials and methods

### Culturing and DNA extraction

*A. faecis* AF1078^T^ (= LMG 28519^T^) and *A. lanthieri* AF1440^T^ (= LMG 28516^T^) type strains, isolated from livestock and human fecal sources, were cultured on modified Agarose Medium (m-AAM) (Oxoid) containing selective antibiotic (cefoperazone, amphotericin-B and teicoplanin) supplements. The plates were incubated at 30 °C under microaerophilic conditions (85% N_2_, 10% CO_2,_ and 5% O_2_) for 3 to 6 days as described previously [22, 23]. Total genomic DNA was extracted and purified using the Wizard Genomic DNA purification kit (Promega, Madison, WI, USA). The concentration of DNA was determined using the Qubit™ 2.0 Fluorometer (Life Technologies, Burlington, ON, Canada). Purified DNA was stored at − 20 °C for further use.

### Genome sequencing

Library preparation and paired-end whole-genome sequencing of *A. faecis* AF1078^T^ and *A. lanthieri* AF1440^T^ reference strains were performed at the National Research Council Canada (Saskatoon, Saskatchewan, Canada). In brief, high-molecular-weight genomic DNA was used as input for library preparation using the Illumina TruSeq DNA library preparation kit (Illumina Inc.) to obtain a library with a median insert size of 300 bp. After PCR enrichment, the resultant library was checked on a Bioanalyzer (Agilent Technologies Inc., Mississauga, ON, Canada) and quantified. The libraries were equimolarly pooled and sequenced on an Illumina HiSeq 2500 platform (Illumina, San Diego, CA, USA), generating 2 × 101 bp paired-end reads for each sequenced fragment. Base-calling and primary quality assessments were performed using the Illumina CASAVA pipeline (v1.8.2).

The mate-pair sequencing was performed at the Ottawa Research and Development Centre, Agriculture and Agri-Food Canada (Ottawa, Ontario, Canada). The mate-paired DNA library was prepared using the Nextera® Mate Pair kit (Illumina, San Diego, CA, USA). DNA fragments with three size ranges (1.8–3.5 Kb, 4.0–7.0 Kb, and 8.0–12.0 Kb) were selected using Pippen SageELF (Sage Scientific, Beverly, MA, USA) and pooled with a mean insert size of 6.1 Kb. The libraries were examined on a Bioanalyzer and then quantified using the KAPA qPCR assay (Wilmington, MA, USA). The sequencing libraries were normalized to 2 nM and then diluted to 6 pM prior to loading on a MiSeq Illumina sequencing platform (Illumina, San Diego, CA, USA), which generated 500 bp mate-paired reads for each sequenced fragment.

### Genome assembly and annotation

Automatic trimming (based on a threshold of Q = 25) using Trimmomatic scanning and de novo assembly using SPAdes genome assembler version 3.11.1 [25] with combined Illumina NextSeq500 data set of paired-end and mate-pair reads for each species was performed. The contigs were assembled into scaffolds using Medusa [26], where *A. nitrofigilis* DSM 7299, *A. butzleri* RM 4018, and *A. cryaerophilus* L406 genomes were used as scaffolding references. GapFiller [27] closed scaffold gaps using raw paired-end sequencing data. Finally, both de novo assembled genomes were annotated with Prokka version 1.12 [28]. The genome sequences and annotations were deposited to the JGI IMG/MER under analysis IDs Ga0136198 (*A. faecis*) and Ga0136182 (*A. lanthieri*) [29].

An Unweighted Pair Group Method with Arithmetic mean (UPGMA) phylogenetic tree was built using the MASH tool version 2.3 with a sketch size of s = 1000, a *k*-mer size of *k* = 21, and 100 random seeds for bootstrap [30]. MASH uses the MinHash technique to assess the pairwise distance between sequences [30]. The bootstrapped phylogenetic tree was visualized using the R package ggtree version 3.2.1 [31]. In this analysis, we included the genomes of strains of nine *Aliarcobacter* species fetched from the National Center for Biotechnology Information (NCBI) database: *A. butzleri* (strain RM 4018: NC_009850.1; strain ED-1: NC_017187.1; strain NCTC 12481^T^: JGI Ga0136182), *A. cibarius* (strain LMG 21996^T^: draft genome NZ_JABW01000000.1; strain H73: NZ_CP043857.1), *A. cryaerophilus* (strain ATCC 43158^T^: NZ_CP032823.1; strain D2610: NZ_CP032825.1), *A. lanthieri* (strain AF 1581: NZ_JARV01000000.1), *A. skirrowii* (strain CCUG 10374^T^: NZ_CP032099.1; strain A2S6: NZ_CP034309.1), *A. thereius* (strain LMG 24486^T^: draft genome NZ_LLKQ01000000.1; strain DU22: draft genome NZ_LCUJ01000000.1), *A. trophiarum* (strain LMG 25534^T^: NZ_CP031367.1; strain CECT 7650: draft genome NZ_PDJS01000000.1), *A. vitoriensis* (strain CECT 9230: draft genome NZ_PDKB01000000.1), as well as more distant members of *Epsilonproteobacteria* in the order *Campylobacterales*: *Helicobacter pylori* (strain 26,695: NC_000915.1), *Campylobacter fetus* (strain 82-40^T^: NC_008599.1), *Sulfurospirillum deleyianum* (strain DSM 6946^T^: NC_013512.1). The tree was rooted in *Wolinella succinogenes* (strain DSM 1740^T^: NC_005090.1).

Gene synteny and homology of *A. faecis* strain AF1078^T^ and *A. lanthieri* strain AF1440^T^ were computed using BLASTp and MCScanX with default parameters (match score ≥ 50, E-value ≤ 10^− 5^, max gaps ≤ 25) [32, 33].

### Genome annotation for pathogenicity assessment

VFanalyzer is an automatic pipeline for a systematic screen of potential virulence factors (VFs) against the Virulence Factor Database (VFDB) [34]. VFanalyzer was used to identify VFs from the predicted genes of *A. faecis* strain AF1078^T^, *A. lanthieri* strain AF1440^T^, *A. butzleri* strain NCTC 12481^T^, *A. cryaerophilus* strain ATCC 43158^T^, *A. skirrowii* strain CCUG 10374^T^, *A. cibarius* strain LMG 21996^T^, *A. thereius* strain LMG 24486^T^, and *A. trophiarum* strain LMG 25534^T^ with default parameters. The genome sequence of the *Campylobacter jejuni* strain NCTC 11168^T^ was used as a reference. Moreover, amino acid sequences related to previously studied virulence factors of *Aliarcobacter* species [35] were collected in a custom database, including *cad*F (Abu_0481), *cj*1349 (Abu_0067), *cia*B (Abu_1549), *irg*A (Abu_0726), *pld*A (Abu_0861), *hec*A (Abu_0940), *hec*B (Abu_0939), *tly*A (Abu_1835), *waa*F (Abu_1800), *waa*C (Abu_1822), *htr*A (Abu_2099), *iam*A (Abu_0107), *fur*1 (Abu_0717), *fur*2 (Abu_1770), *lux*S (Abu_0111), *ure*B (Abu_0807), *ure*D (Abu_0805), *ure*E (Abu_0808), *ure*G (Abu_0810), *fla*A (Abu_2254), *fla*B (Abu_2255), *flg*H (Abu_0208), *mot*A (Abu_0271) and *mvi*N (Abu_0878) from *A. butzleri* strain RM4018^T^ (GenBank assembly accession: GCA_000014025.1), and *iro*E (AA20_05105) from *A. butzleri* strain L348 (GenBank assembly accession: GCA_001010585.1), and *vir*F (AAX29_00642) from *A. thereius* strain DU22 (GenBank assembly accession: GCA_001695335.1) .

TA system is a set of genes encoding a pair of stable toxin and unstable anti-toxin. TAfinder was used to predict type II TA loci in *A. faecis* strain AF1078^T^ and *A. lanthieri* strain AF1440^T^ with default parameters [36].

The VF and TA genes in the complete genome of *A. faecis* AF1078^T^ and *A. lanthieri* AF1440^T^ strains were visualized using the circlize package in R [37].

### PCR-based assays for validation of virulence, antibiotic resistance, and toxin (VAT) genes

The detection of VAT genes was carried out using our previously developed species- and gene-specific primer pairs, mono- and multiplex Polymerase Chain Reaction (PCR) protocols [38]. For *A. lanthieri*, a total of 11 including six virulence (*cad*F, *cia*B, *irg*A, *mvi*N, *pld*A, and *tly*A), two antibiotic resistance [*tet*(O) and *tet*(W)] and three cytolethal distending toxin (*cdt*A, *cdt*B, and *cdt*C) genes were tested. However, six virulence (*cad*F, *cia*B *irg*A, *mvi*N, *pld*A and *tly*A), two antibiotic resistance [*tet*(O) and *tet*(W)] and two cytolethal distending toxin (*cdt*A and *cdt*C) genes were tested for *A. faecis*. No *ctd*B-based PCR assay was available for *A. faecis*.

The amplicon sizes of each mono- and multiplex PCR reaction were confirmed by 2.5% agarose gel electrophoresis (Fisher Scientific) using a 100 bp DNA size marker (Life Technologies, Grand Island, NY). The agarose gels were stained in ethidium bromide (0.5 μg/mL), and Alpha Imager (Fisher Scientific) was used for scanning and documentation.

## Results and discussion

### General features of *a. faecis* and *A. lanthieri* genomes

**Fig. 1:**
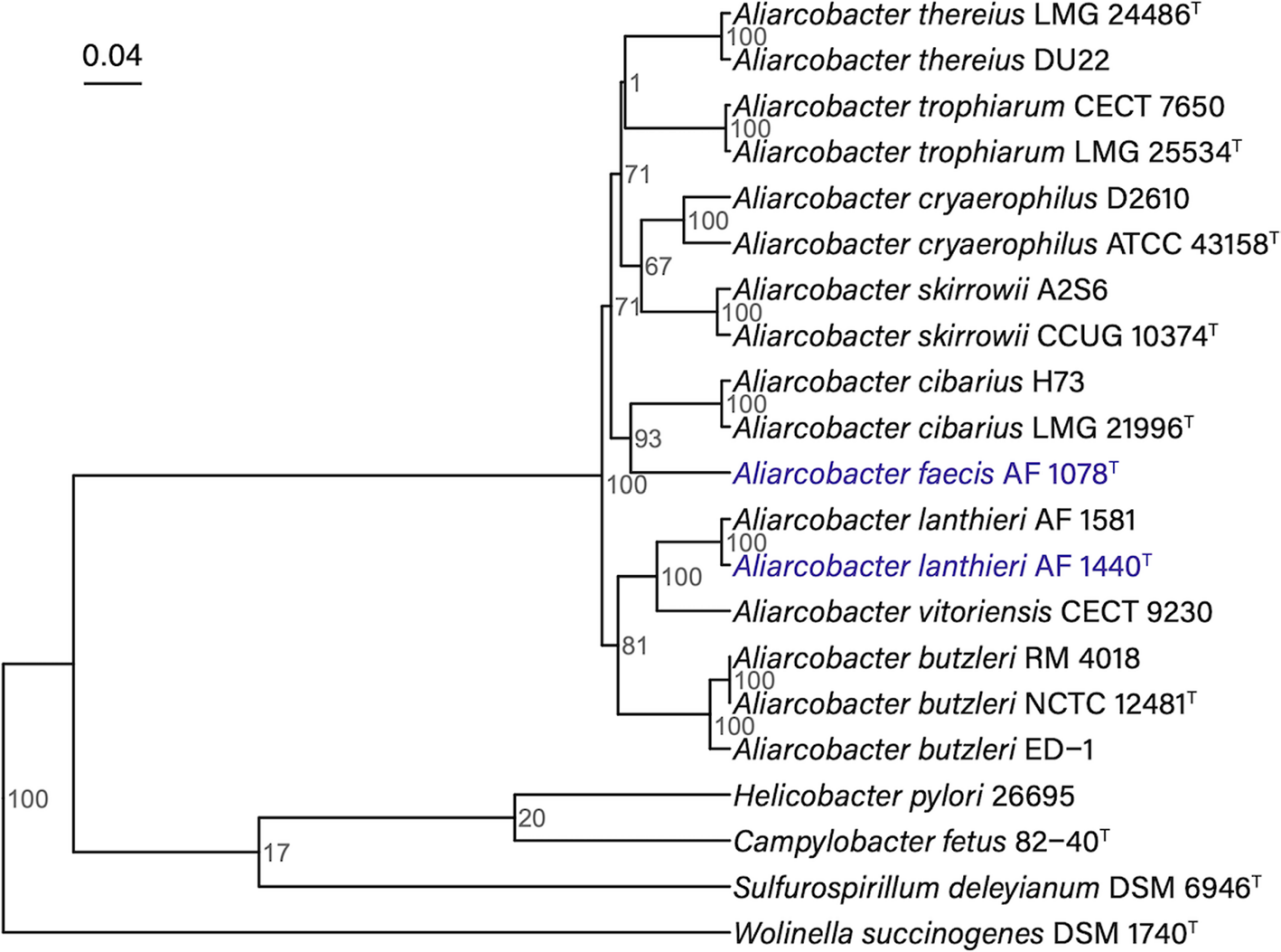
A comparable Mash-based tree generated from whole genomes. The UPGMA phylogenetic tree was constructed using the MinHash algorithm with 100 bootstraps where distances were calculated from whole genome sequences. The scale bar represents the Mash distance

The genome of *A. faecis* AF1078^T^ (= LMG 28519^T^) reference strain contained 2,327,155 bp in one scaffold, and the genome of *A. lanthieri* AF1440^T^ (= LMG 28516^T^) reference strain contained 2,234,737 bp in one scaffold. The overall GC contents of the two genomes were 27.0 and 26.4%, respectively, which were consistent with other *Arcobacter* *sensu* *lato* species (Table 1). The numbers of protein-coding genes predicted in *A. faecis* and *A. lanthieri* genomes were 2319 and 2230, respectively (Table 1). *A. lanthieri* and *A. faecis* had a similar percentage (73%) of functionally annotated protein-coding genes with Clusters of Orthologous Groups (COGs) (Table 1). The phylogenetic tree of whole genome comparison shows that the strains of the two new species clustered with *A. vitoriensis* and *A. cibarius*, respectively (Fig. 1). This confirms previous maximum-likelihood phylogenetic analysis based on 16S rRNA and housekeeping genes [22, 23].

**Table 1:** Statistical summary of assembly and annotation of reference genomes of *Aliarcobacter lanthieri and A. faecis*

	***A. lanthieri*** AF1440^**T**^		***A. faecis*** AF1078^**T**^	
	No.	%	No.	%
DNA, total number of bases	2,234,737	100	2,327,155	100
DNA coding number of bases	2,109,823	94.41	2,180,685	93.71
DNA G + C number of bases	589,766	26.39	627,419	26.96
Genes total number	2268	100	2351	100
Protein coding genes	2230	98.32	2319	98.64
Genes with function prediction	1749	77.12	1745	74.22
Protein coding genes with COGs	1491	65.74	1484	63.12
COG clusters	1097	73.57	1086	73.18

Table 2 shows the number of predicted genes associated with the COG functional categories (E-value < 10^− 10^) in the complete reference genomes of *Aliarcobacter* species. To avoid errors in gene copy number estimation, draft genomes of *A. cibarius* and *A. thereius* were not included. Overall, the genes were assigned to 23 out of 26 COG categories in the genomes of all *Aliarcobacter* species, as shown in Table 2. Genes encoding bacterial Type II (*pul*E, *pul*F) and III (*fli*F, *fli*N, *ylq*H) secretory pathways were identified from genomes of all strains (Table S1). Genes encoding bacterial Type IV (*vir*B4), VI (*dot*U, *vas*A, *vas*K, *tss*A, *vir*G) and VII (*hcp*) protein secretion systems were identified in the reference strains of *A. faecis* but not in those of *A. lanthieri* or *A. butzleri* included in this study (Table S1). However, a study found a full Type IV pathway for a particular *A. butzleri* strain D4963 [39], suggesting significant genetic variation between strains isolated from different geographical locations. Besides, genes (*tat*A, *tat*B, and *tat*C) involved in the Twin-arginine translocation (Tat) secretion pathway were found in *A. faecis*, *A. lanthieri* and *A. butzleri* (Table S1). Furthermore, the mobilome COG category (code X) was underrepresented in the reference genome of *A. lanthieri*, carrying only one gene associated with COG2932 from that group (Table 2). *A. faecis* and *A. butzleri* had 11 and seven genes, respectively, related to COG code X (Table 2). The presence of the mobile genetic elements, such as prophages and transposon, may suggest horizontal gene transfer of potentially antimicrobial resistance and/or adaptation genes.

**Table 2:** Number of predicted orthologous genes associated with COG functional categories in *Aliarcobacter* species

COG Category	***A. faecis*** AF1078^**T**^	***A. lanthieri*** AF1440^**T**^	***A. butzleri*** NCTC 12481^**T**^	***A. cryaerophilus*** ATCC 43158^**T**^	***A. skirrowii*** CCUG 10374^**T**^	***A. trophiarum*** LMG 25534^**T**^	Description
A	0	0	0	0	0	0	RNA processing and modification
B	0	0	0	0	0	0	Chromatin structure and dynamics
C	100	90	110	117	119	125	Energy production and conversion
D	18	15	16	30	26	24	Cell cycle control, cell division, chromosome partitioning
E	116	126	139	149	143	145	Amino acid transport and metabolism
F	56	53	57	63	62	62	Nucleotide transport and metabolism
G	41	46	52	63	62	63	Carbohydrate transport and metabolism
H	117	115	108	127	126	122	Coenzyme transport and metabolism
I	49	49	47	63	50	59	Lipid transport and metabolism
J	155	160	161	183	177	177	Translation, ribosomal structure and biogenesis
K	81	78	92	76	77	65	Transcription
L	80	78	71	107	106	87	Replication, recombination and repair
M	123	133	141	167	129	149	Cell wall/membrane/envelope biogenesis
N	33	34	35	56	53	49	Cell motility
O	65	72	76	98	95	100	Posttranslational modification, protein turnover, chaperones
P	118	142	118	107	119	113	Inorganic ion transport and metabolism
Q	9	6	7	13	10	9	Secondary metabolites biosynthesis, transport and catabolism
R	99	100	104	130	128	118	General function prediction only
S	36	29	39	41	51	36	Function unknown
T	133	117	143	185	156	155	Signal transduction mechanisms
U	38	25	22	45	57	44	Intracellular trafficking, secretion, and vesicular transport
V	43	34	41	46	43	36	Defense mechanisms
W	2	2	2	6	4	3	Extracellular structures
X	11	1	7	11	13	31	Mobilome: prophages, transposons
Y	0	0	0	0	0	0	Nuclear structure
Z	2	2	2	3	4	3	Cytoskeleton

### Detection and comparison of virulence-associated genes of *Aliarcobacter* species

**Fig. 2:**
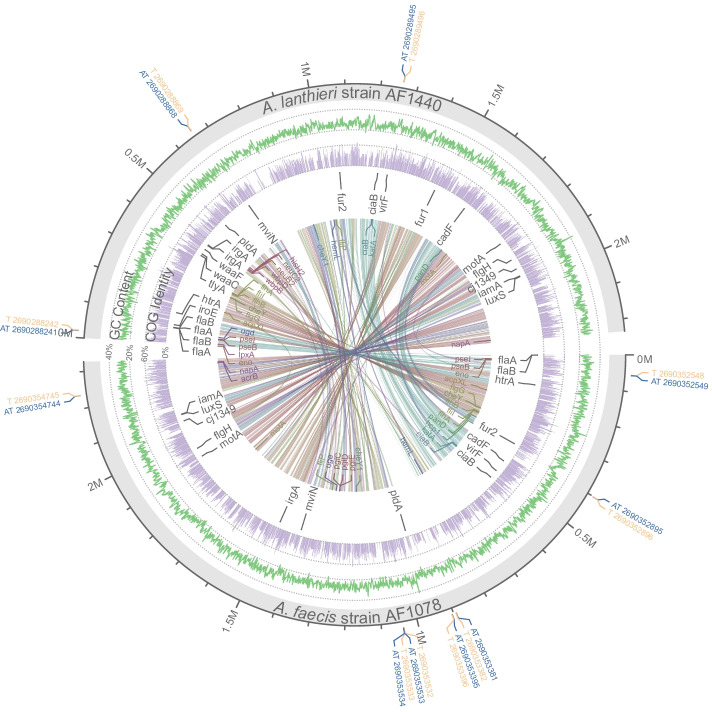
Circular plot of genomes of *A. lanthieri* AF1440^T^ and *A. faecis* AF1078^T^ reference strains. From outward to inward: Circle 1: Toxin genes shown in orange and anti-toxin genes in blue; Circle 2: GC content of the genomes; Circle 3: Predicted genes associated with COG functional categories; Circle 4: Virulence factors (VF) identified from *Aliarcobacter* and *Campylobacter* species; Circle 5: VF genes annotated by VFanalyzer, and the colors represent VF classes. The inner links indicate collinear genes of both genomes estimated from the gene synteny analysis

Twenty-six virulence-associated genes were previously reported in *A. butzleri* and other *Aliarcobacter* species [15, 35]. This study compared the 26 genes and identified 15 in *A. faecis* and 20 in *A. lanthieri* (Table 3, Fig. 2). Besides, additional putative VF and TA genes of *A. faecis* and *A. lanthieri* were identified using VFanalyzer and TAfinder (Table 4; Fig. 2) against the VFDB and TADB databases, respectively [34, 36]. Other known and putative zoonotic pathogens in the genus *Aliarcobacter* were also annotated using VFanalyzer (Table 4). The E-values of putative VFs were < 10^− 10^, and in general, the coverages were > 90% (Table 4). Here, we present these genes into functional categories, including motility and export apparatus, invasion and stress resistance, adherence, antimicrobial resistance, TA systems, and general resistance.

**Table 3:** Presence and copy numbers of known virulence factors in *A. faecis* AF1078^T^ and *A. lanthieri* AF1440^T^

Gene	COG Category	COG ID	***A. faecis*** AF1078^**T**^	***A. lanthieri*** AF1440^**T**^	Product
*tly*A	J	COG1189	–	1	23S rRNA (cytidine1920–2′-O)/16S rRNA (cytidine1409–2′-O)-methyltransferase
*vir*F	K	COG2207	1	1	AraC-type DNA-binding protein
*cad*F	M	COG2885	1	1	OmpA-OmpF porin, OOP family
*iam*A	M	COG1127	1	1	phospholipid/cholesterol/gamma-HCH transport system ATP-binding protein
*mvi*N	M	COG0728	1	1	putative peptidoglycan lipid II flippase
*pld*A	M	COG2829	1	1	phospholipase A1
*waa*C	M	COG0859	–	1	heptosyltransferase-1
*waa*F	M	COG0859	–	1	heptosyltransferase-2
*fla*A	N	COG1344	1	2	flagellin
*fla*B	N	COG1344	1	2	flagellin
*flg*H	N	COG2063	1	1	flagellar L-ring protein precursor FlgH
*htr*A	O	COG0265	1	1	serine protease Do
*fur*1	P	COG0735	–	1	Fur family transcriptional regulator, ferric uptake regulator
*fur*2	P	COG0735	1	1	Fur family transcriptional regulator, ferric uptake regulator
*irg*A	P	COG4771	1	2	outer membrane receptor for ferrienterochelin and colicins
*iro*E	R	COG2819	–	1	Predicted hydrolase of the alpha/beta superfamily
*lux*S	T	COG1854	1	1	S-ribosylhomocysteine lyase /quorum-sensing autoinducer 2 (AI-2) synthesis protein LuxS
*cia*B	–	–	1	1	*Campylobacter* invasion antigens; involved in secretion of virulence factors thorough flagellum
*cj*1349	–	–	1	1	Fibronectin-binding protein A N-terminus (FbpA)
*mot*A	–	–	1	1	MotA/TolQ/ExbB proton channel family protein
*hec*A	–	–	–	–	Adhesive
*hec*B	–	–	–	–	Adhesive
*ure*B	–	–	–	–	Urease gene
*ure*D	–	–	–	–	Urease gene
*ure*E	–	–	–	–	Urease gene
*ure*G	–	–	–	–	Urease gene

**Table 4:** Virulence factors annotated with Vfanalyzer in *Aliarcobacter* species. The copy number and the BLASTp statistics of the genes with the highest percent of identity are shown in the format of “copy number (E-value | % identity | % coverage)”. A minus sign “-” represents “not detected”

Class	Virulence Factors	Gene	***A. faecis*** AF1078^**T**^	***A. lanthieri*** AF1440^**T**^	***A. butzleri*** NCTC 12481^**T**^	***A. cryaerophilus*** ATCC 43158^**T**^	***A. skirrowii*** CCUG 10374^**T**^	***A. thereius*** LMG 24486^**T**^	***A. cibarius*** LMG 21996^**T**^	***A. trophiarum*** LMG 25534^**T**^
Acid resistance	Urease	*ure*A	–	–	1 (1.14e-80|54|98)	–	–	–	–	–
		*ure*B	–	–	1 (0|65|100)	–	–	–	–	–
		*ure*G	–	–	1 (8.13e-80|60|97)	–	–	–	–	–
Adherence	LPS O-antigen	*his*F2	–	–	2 (8.13e-99|53|97)	–	–	–	–	–
		*his*H2	–	1 (1.03e-77|51|99)	–	–	–	–	–	–
		*wbp*B	–	1 (8.68e-116|55|97)	1 (2.2e-111|56|93)	–	–	–	–	–
		*wbp*D	–	1 (8.26e-70|53|93)	1 (3.37e-70|53|93)	–	–	–	–	–
		*wbp*E	–	–	–	–	–	–	1 (4.52e-131|50|99)	–
		*wbp*G	–	–	1 (3.52e-175|61|100)	–	–	–	–	–
		*wbp*H	–	–	1 (7.68e-159|56|99)	–	–	–	–	–
		*wbp*I	–	–	1 (1.17e-156|60|99)	–	–	–	–	–
		Undetermined	–	–	–	–	–	–	1 (3.69e-147|59|99)	–
Antiphagocytosis	Capsule	*ugd*	–	1 (0|73|100)	–	–	–	–	–	–
		*uge*	1 (2.88e-150|57|98)	–	1 (2.6e-158|59|98)	–	–	–	1 (7.36e-148|56|99)	–
		Undetermined	1 (0|71|99)	1 (9.12e-65|53|92)	1 (0|72|100)	3 (0|71|100)	3 (0|72|100)	1 (7.17e-69|55|92)	2 (0|73|97)	–
Chemotaxis and motility	Flagella	*che*Y	1 (2.2e-51|67|98)	1 (2.92e-50|66|98)	1 (2.62e-50|66|98)	–	1 (2.99e-51|66|98)	1 (3.84e-50|64|98)	–	–
Colonization and Immune evasion	Capsule biosynthesis and transport	Undetermined	1 (1.7e-105|50|98)	1 (1.3e-136|53|98)	–	1 (3.35e-103|50|98)	1 (2.54e-102|51|98)	1 (3.39e-108|51|97)	2 (2.08e-137|52|99)	1 (1.91e-102|50|98)
Efflux pump	AcrAB	*acr*B	1 (0|53|98)	–	–	–	–	–	–	–
		Undetermined	1 (0|51|97)	1 (0|53|98)	2 (0|55|100)	1 (0|52|98)	1 (0|53|100)	1 (0|52|98)	1 (0|53|98)	1 (0|52|98)
Endotoxin	LOS	*lpx*A	–	1 (5.51e-84|50|95)	–	–	–	–	–	–
Enzyme	Streptococcal enolase	*eno*	1 (2.04e-156|54|99)	1 (3.68e-156|54|99)	1 (4.67e-160|55|99)	1 (8.53e-157|55|97)	1 (1.23e-157|56|96)	1 (1.98e-157|54|99)	1 (7.89e-156|54|99)	1 (9.52e-157|55|99)
Glycosylation system	N-linked protein glycosylation	*pgl*C	1 (6.18e-90|61|99)	–	–	–	–	–	–	–
		*pgl*D	1 (1.22e-53|51|96)	–	–	–	–	–	–	–
		*pgl*E	1 (8.61e-162|59|99)	–	–	–	–	–	–	–
	O-linked flagellar glycosylation	*neu*B2	–	1 (2.77e-155|65|100)	–	1 (2.09e-149|61|100)	–	–	1 (1.32e-155|65|100)	1 (4.43e-156|62|100)
		*neu*C2	–	1 (5.03e-139|52|99)	–	1 (6.19e-142|49|99)	–	–	–	1 (5.99e-139|48|99)
		*pse*B	1 (0|73|99)	1 (2.4e-180|72|99)	1 (0|75|99)	1 (0|75|99)	1 (0|75|100)	1 (1.04e-180|74|99)	–	–
		*pse*I	1 (2.45e-133|56|99)	1 (1.97e-132|56|99)	1 (9.3e-133|56|100)	1 (1.84e-134|56|98)	1 (4.77e-134|57|99)	1 (5.77e-134|56|99)	–	–
		*ptm*B	–	–	–	1 (8.15e-93|60|97)	–	–	1 (8.29e-99|62|99)	–
Immune evasion	Capsule	Undetermined	2 (0|63|100)	4 (0|75|99)	2 (0|66|100)	3 (0|77|99)	1 (1.82e-80|54|96)	1 (7.15e-82|53|94)	3 (0|76|99)	3 (0|77|99)
	Exopolysaccharide	*gal*E	–	–	–	1 (3.7e-152|60|99)	1 (1.98e-156|62|99)	1 (5.82e-157|62|99)	1 (3.01e-154|60|99)	1 (1.22e-153|62|97)
	Hyaluronic acid (HA) capsule	*gal*U	–	–	1 (7.19e-92|51|93)	–	1 (2.27e-88|50|95)	1 (1.65e-87|50|93)	–	1 (2.89e-92|50|95)
	LOS	Undetermined	2 (1.78e-152|70|98)	9 (5.36e-177|81|98)	7 (6.19e-160|72|98)	10 (9.78e-175|79|98)	13 (2.09e-178|80|98)	7 (1.71e-85|66|96)	8 (5.61e-179|81|98)	8 (2.98e-158|71|99)
	LPS	*acp*XL	1 (6.99e-22|60|89)	1 (7.32e-23|62|89)	1 (7.4e-23|62|89)	1 (2.34e-22|60|89)	1 (7.9e-23|62|89)	1 (3.04e-22|60|89)	1 (6.99e-22|60|89)	1 (2.76e-23|62|89)
	LPS	*fab*Z	–	–	1 (6.49e-44|50|97)	–	–	–	–	–
Immune modulator	Neutrophil-activating protein (HP-NAP)	*nap*A	1 (3.17e-53|50|99)	1 (2.3e-56|54|99)	1 (4.43e-56|55|97)	1 (9.86e-51|51|98)	1 (1.11e-48|50|98)	1 (1.78e-50|51|99)	–	–
Invasion	Campylobacter invasion antigen	*cia*B	1 (8.94e-148|40|97)	1 (6.41e-147|40|99)	1 (2.14e-147|40|97)	1 (1.8e-144|39|98)	1 (1.38e-147|41|97)	1 (3.6e-153|41|97)	1 (2.79e-149|41|97)	1 (1.29e-140|39|97)
	Flagella	*che*Y1	1 (1.81e-30|51|93)	1 (1.23e-28|50|91)	1 (3.02e-31|52|91)	1 (7.03e-29|50|91)	–	–	1 (2.02e-29|51|93)	1 (3.71e-30|51|91)
Iron uptake	Heme biosynthesis	*hem*L	1 (3.29e-161|53|99)	1 (1.94e-161|54|97)	1 (4.8e-159|52|99)	1 (2.76e-159|53|99)	1 (6.7e-162|53|97)	1 (3.72e-164|53|99)	1 (2.06e-164|53|99)	1 (1.65e-158|52|99)
Lipid and fatty acid metabolism	Pantothenate synthesis	*pan*D	1 (4.36e-34|51|91)	1 (1.04e-36|55|91)	1 (1.31e-36|52|94)	1 (9.87e-35|53|88)	1 (1.99e-34|50|88)	1 (1.2e-35|52|88)	1 (4.91e-35|53|88)	1 (4.36e-35|53|88)
Motility and export apparatus	Flagella	*flg*G	1 (5.01e-100|58|100)	1 (2.15e-98|58|100)	1 (4.53e-101|57|100)	1 (1.09e-100|59|100)	1 (4.07e-100|60|100)	1 (7.31e-102|60|100)	1 (4.85e-100|56|100)	1 (2.52e-103|60|100)
		*flh*A	1 (0|50|98)	1 (0|50|98)	1 (0|50|98)	1 (0|50|98)	1 (0|51|98)	1 (0|49|98)	1 (0|50|98)	1 (0|50|99)
		*flh*B	1 (7.69e-103|46|100)	1 (1.28e-112|48|98)	1 (1.37e-102|45|99)	1 (3.34e-111|49|98)	1 (6.79e-104|47|98)	–	1 (1.52e-103|45|99)	1 (3.19e-107|48|98)
		*fli*I	1 (1.09e-173|56|100)	1 (9.59e-176|57|97)	1 (3.54e-176|61|94)	1 (2.85e-176|56|100)	1 (1.19e-179|57|97)	1 (3.05e-179|58|97)	1 (3.43e-172|59|94)	1 (2.19e-180|57|100)
		*fli*P	1 (7.73e-103|62|92)	1 (3.34e-104|62|100)	1 (1.71e-103|61|95)	1 (4.16e-102|57|100)	1 (8.62e-106|59|100)	1 (1.14e-104|59|100)	1 (5.84e-102|62|92)	1 (1.33e-104|59|100)
		*mot*A	1 (6.04e-96|56|97)	1 (1.4e-88|53|99)	1 (9.92e-94|54|98)	1 (9.76e-88|54|98)	1 (1.12e-86|52|98)	1 (1.05e-87|53|98)	1 (1.98e-96|57|98)	1 (9.14e-89|59|96)
		Undetermined	1 (6.28e-41|55|99)	1 (6.42e-43|56|98)	1 (9.66e-37|52|92)	1 (1.23e-41|56|98)	1 (3.61e-46|58|100)	1 (4.96e-43|57|99)	1 (2.79e-42|55|98)	1 (3.67e-42|57|99)
Nutritional virulence	Pyrimidine biosynthesis	Undetermined	1 (0|51|100)	1 (0|51|100)	1 (0|54|100)	1 (0|52|100)	1 (0|51|100)	1 (0|52|100)	1 (0|51|100)	1 (0|51|100)
Secretion system	T6SS	*hcp*1	–	–	–	–	1 (1.14e-68|57|96)	–	–	–
		Undetermined	1 (0|64|100)	–	–	–	1 (0|64|100)	–	–	–
	T6SS-1	Undetermined	1 (3.28e-56|55|94)	–	–	–	1 (1.85e-55|55|95)	–	–	–
	T7SS	*hcp*1	1 (1.24e-73|59|96)	–	–	–	–	–	–	–
Serum resistance and immune evasion	LPS	*wbt*I	–	–	1 (8.93e-178|69|96)	–	–	–	–	1 (0|69|97)
		Undetermined	2 (0|71|100)	2 (0|65|100)	1 (0|52|100)	1 (0|68|99)	1 (0|72|99)	–	2 (0|73|100)	1 (0|51|99)
Stress adaptation	Catalase	*kat*A	1 (0|57|99)	1 (0|57|99)	–	1 (0|58|99)	1 (0|58|99)	1 (0|58|99)	–	1 (0|56|99)
	Catalase-peroxidase	*kat*G	–	–	1 (0|60|99)	–	–	–	–	–

### Motility and export apparatus

Bacterial flagellum can affect its virulence by determining the physical motility and act as a secretion system for other virulence factors [40]. Flagella genes *fla*A, *fla*B, *flg*G, *flh*A, *flh*B, *fli*I, *fli*P, *che*Y1, and *mot*A were found in both *A. faecis* and *A. lanthieri*, reference strain genomes (Tables 3 and 4). The flagellum apparatus of pathogenic bacteria is considered a secretion system composed of flagellar proteins, which forms a needle to inject bacterial toxins into the host cell. For example, it was reported that the flagellum of *H. pylori* is required to colonize the mucosal membrane of the stomach as opposed to penetrating the gastric mucosa [41]. Comparative genomics analysis also claimed that some non-flagellum type III secretion systems were evolved from flagellar secretion systems through a series of genetic deletions, innovations, and recruitments of components from other cellular structures [42].

### Invasion and stress resistance

Orthologs of virulence factors *cia*B, *iam*A, and *mvi*N were detected in the genomes of both *A. faecis* and *A. lanthieri* reference strains. These genes provide pathogens a competitive advantage to survive in the bacterial community (Table 3). Gene *cia*B encodes one of the invasion antigens (Cia proteins), deletion of which resulted in significantly attenuated virulence in *C. jejuni* [43]. In addition, it has been suggested that flagellum serves as an export apparatus or secretion channel for Cia proteins [43]. Studies showed that mutants of *Yersinia* and *C. jejuni* without functional flagellar apparatus lack the ability to secrete Cia proteins in comparison to wild type [43]. Another secretion-associated gene, *mvi*N, encodes peptidoglycan (a.k.a murein) flippase. Murein protects the gram-negative bacterial cell membrane from osmotic stress and serves as an anchor for virulence factors [44, 45]. The murein layer is vitally important for bacterial cells’ survival and is shown in Table 3. It shows that *A. faecis* and *A. lanthieri* carry a single copy of *mvi*N ortholog.

Similarly, orthologs of *iam*A and *pld*A were found in both *A. faecis* and *A. lanthieri* genomes (Table 3). Of these, *iam*A, an invasion-associated marker gene, was also found in *C. jejuni* and reported to be associated with diarrhea [46, 47]. Previous studies showed that the PLA activity in *Legionella* spp., *E. coli* and *Mycoplasma hyorhinis* was associated with the impairment of host intestine cell membranes through hydrolyzation [48]. The lysis property of PLA also helps bacteria to acquire iron from erythrocytes by penetrating the host cell membranes [49]. Orthologs of *waa*C and *waa*F were discovered in *A. lanthieri* but not in *A. faecis* (Table 3). These two genes were also virulence determinants involved in the biosynthesis of liposaccharide in *A. thereius* and other species of the family *Campylobacteraceae* [50].

The orthologs of *irg*A and *fur*2 were identified in both *A. faecis* and *A. lanthieri* genomes (Table 3). It was previously suggested that *irg*A, the enterobactin receptor gene, is induced by low iron, and the regulation depends on the iron-responsive master regulator Fur [51]. In addition, *irg*A ortholog was described for *A. butzleri* [15] and to a lesser degree to some *Campylobacter* species [52].

Furthermore, the urease enzyme secreted by bacteria promotes its own persistence in the stomach, allowing them to quickly migrate into the gastric mucosal epithelial line by chemotaxis, where pH is comparatively higher [53]. Although the urease enzyme gene cluster was found along with some accessory genes (*ure*B, *ure*D, *ure*E, and *ure*G) in *A. butzleri* (Table 3) [39], it is not identified in *A. faecis* and *A. lanthieri* genomes (Table 3).

### Adherence

Adherence mechanisms of bacterial pathovars play a major role in invading the hosts and competing with intestinal commensals [54]. Cell surface adhesion encoding genes represented by orthologs of *cad*F, *hec*A, and *cj*1349 have been considered crucial VFs for pathogenic bacteria [55]. Table 3 indicates that only *cad*F and *cj*1349 were are detected, whereas *hec*A was not found in the genome of either species.

Adhesin encoded by *cad*F was found to mediate binding to Fibronectin, a protein present on the surface of epithelial cells [56]. Studies have also shown that *Campylobacter* cells lacking *cad*F exhibited a 50–90% reduction in adherence to epithelial cells [56]. Both proteins, *cad*F and *cj*1349c, were important for *C. jejuni* to adhere to the outer membrane of chicken cells and increase their virulence [56]. Orthologs of *hec*A/*hec*B, previously detected exclusively in a few strains [56], were not found in *A. faecis* and A*. lanthieri* genomes (Table 3). Although not all known adherence genes were found in the reference genomes of *A. faecis* and A*. lanthieri* (Table 3 and Table 4), these two species may still exhibit adherence ability linking to pathogenicity.

### Antimicrobial resistance

Antimicrobial resistance genes consist of the most abundant group of virulence-related factors. Genomes of reference strains of *A. faecis, A. lanthieri*, *A. butzleri*, and other *Aliarcobacter* species contain efflux pumps associated with antibiotic resistance (Table 4). The identified pumps belong to the Resistance Nodulation cell Division (RND) protein superfamily, one of the most studied antiporters found in bacteria [57]. The identified genes were highly similar to *acr*B*,* encoding a multidrug efflux pump [58]. *Acr*B is a well-described antiporter involved in resistance to lipophilic β-lactam antibiotics, such as carbapenems and cephalosporins, fluoroquinolones, tetracyclines (including tigecycline), chloramphenicol, macrolides, trimethoprim, ethidium, rifampicin, and novobiocin [58]. It is of particular interest as previous studies showed that *A. butzleri* strains exhibited resistance to a variety of antibiotics, where the majority of them belong to β-lactams and some to quinolones and coumarins [15].

**Fig. 3:**
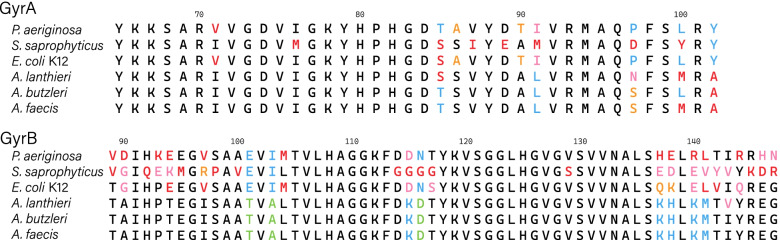
Amino acid sequence alignment of the gyrase subunit A and B (*gyr*A, *gyr*B) gene from *A. lanthieri* AF1440^T^, *A. faecis* AF1078^T^, *A. butzleri* NCTC 12481^T^, *Escherichia coli* K12, *Pseudomonas aeruginosa* ATCC 27853 (susceptible to quinolones), and *Staphylococcus saprophyticus* ATCC 15305 (resistant to novobiocin) reference strains

As indicated above, RND transporters like *Acr*B may determine resistance to quinolones and coumarins [58, 59]. This class of antibiotics targets bacterial DNA gyrase, type II topoisomerase, which plays an essential role in DNA replication [60]. However, significant data accumulated suggests that the resistance to such antibiotics may be acquired through specific mutations in the DNA gyrase gene [19, 20]. According to Vickers [20], resistance to novobiocin (coumarin antibiotic) is acquired through two amino acid residue mutations G(80) K and L(140) R in the B-subunit of DNA gyrase (*gyr*B) gene in *Staphylococcus saprophyticus*. Alignment of *gyr*B gene from novobiocin susceptible strain of *Staphylococcus saprophyticus* with homologs from *A. faecis*, *A. lanthieri*, and *A. butzleri* showed that these *Aliarcobacter* species carry A(80) and R(140) residues in *gyr*B gene (Fig. 3). This may indicate partial resistance to novobiocin due to *gyr*B mutations.

Subunit-A of DNA gyrase (*gyr*A) may also define resistance to quinolones. According to a previous study, the mutations of two amino acid residues in *gyr*A, T(83) and D(87) are enough to gain resistance to a variety of quinolones [61]. The alignment of *gyr*A genes of five *Arcobacter* strains with its orthologs in *Pseudomonas aeruginosa* strain ATCC 27853, susceptible to quinolones, showed that residues T(83) and D(87) marked on *P. aeruginosa* sequence remain intact for most strains including *A. butzleri* (Fig. 3). On the other hand, *A. lanthieri* showed Serine at position 83 instead of Threonine, which still indicates susceptibility to quinolones [61]. As shown in previous studies, *A. butzleri* is susceptible to a high concentration of quinolones, much higher than those determined by mutations in gyrase [15, 62]. Thus, it is suggested that RND transporters are the main contributors to quinolones resistance in *Aliarcobacter* species, making Gyrase mutations less significant.

### Toxin-antitoxin (TA) systems

TA system is a pair of genes encoding a toxin and its cognate anti-toxin, and it helps bacteria withstand lethal antibiotic exposure or environmental stresses [63]. We identified seven TA systems in *A. faecis* and three in *A. lanthieri* (Table 5). The *Tet*R-type transcriptional regulator is located near a gene encoding a major facilitator superfamily (MFS) efflux transporter (Table 5), showing the resistance to disinfectants of quaternary ammonium compounds (QACs), including benzalkonium chloride (BAC) [64]. Also, *hip*BA TA systems are present in both species (Table 5). The *hip*B anti-toxin neutralizes the HipA toxin, a serine/threonine kinase inhibiting cell growth where *hip*BA modules are found in divergent bacterial genomes, and many are related to the persistence of antibiotic resistance [63].

**Table 5:** Toxin-antitoxin systems annotated with TAfinder in *A. faecis* AF1078^T^ and *A. lanthieri* AF1440^T^

Genome	JGI Gene ID	Product	Toxin/Anti-toxin	Domain Annotation
*A. faecis* AF1078^T^	2,690,352,548	MFS transporter, DHA1 family, bicyclomycin/chloramphenicol resistance protein	Toxin	pfam12568
	2,690,352,549	transcriptional regulator, TetR family	Anti-toxin	pfam00440
	2,690,352,895	HTH-type transcriptional regulator / antitoxin HigA	Antitoxin	COG5499; Xre like domain
	2,690,352,896	mRNA interferase HigB	Toxin	COG4680; relE like domain
	2,690,353,381	hypothetical protein	Anti-toxin	cd00093
	2,690,353,382	serine/threonine-protein kinase HipA	Toxin	COG3550
	2,690,353,395	DNA-binding response regulator, the OmpR family, contains REC and winged-helix (wHTH) domain	Anti-toxin	smart00862
	2,690,353,396	hypothetical protein	Toxin	TIGR03694
	2,690,353,532	hypothetical protein	Toxin	COG4891
	2,690,353,533	Transglutaminase-like superfamily protein	Antitoxin	pfam01047
	2,690,353,533	Transglutaminase-like superfamily protein	Toxin	COG1246
	2,690,353,534	RNA polymerase sigma-70 factor, ECF subfamily	Anti-toxin	cd06171
	2,690,354,744	Putative addiction module component	Anti-toxin	RHH like domain
	2,690,354,745	ParE toxin of type II toxin-antitoxin system, parDE	Toxin	relE like domain
*A. lanthieri* AF1440^T^	2,690,288,241	transcriptional regulator, TetR family	Antitoxin	pfam00440
	2,690,288,242	MFS transporter, DHA1 family, bicyclomycin/chloramphenicol resistance protein	Toxin	pfam12568
	2,690,288,868	Helix-turn-helix domain-containing protein	Anti-toxin	COG5606; Xre like domain
	2,690,288,869	serine/threonine-protein kinase HipA	Toxin	COG3550; HipA like domain
	2,690,289,495	AraC-type DNA-binding protein	Anti-toxin	Xre like domain
	2,690,289,496	putative acetyltransferase	Toxin	GNAT like domain

In *A. lanthieri*, the AraC-type DNA-binding protein, which regulates the expression of the proteins requiring the sugar L-arabinose, is adjacent to a putative acetyltransferase (Table 5) conserved in most environmental mycobacterial species, such as *Mycobacterium smegmatis* [65].

On the other hand, in *A. faecis*, the HigB/HigA TA system was found (Table 5), which regulates VFs pyochelin, pyocyanin, swarming, and biofilm formation in *Pseudomonas aeruginosa* [66]. Besides, the ParDE TA system was also identified. This TA system helps bacteria resist heat and antibiotics [67]. We also found a TA system in *A. faecis* related to the OmpR family DNA binding response regulator and a putative gene of acyltransferase (Table 5). The OmpR protein was found to regulate the expression of a type III secretion system at the transcriptional level in Enterohemorrhagic *E. coli* [68].

### General resistance

Conservatively, general resistance factors are not VFs. They determine overall cell stability as part of the housekeeping processes. We identified five chaperone genes, *clp*A*, clp*B*, gro*EL, *dna*K and EF-Tu, as general resistance factors in *A. faecis*, *A. lanthieri*, and *A. butzleri*, which previously showed a connection to bacterial virulence.

The main function of chaperones is protein folding, and it might determine cell resistance against abiotic stress [69]. In particular, genes *clp*A and *clp*B, encoding members of the Hsp100/Clp ATPases family in chaperones, were found necessarily required for intracellular multiplication and heat tolerance [70]. These chaperones, identified as a part of the Clp proteolytic complex, were first reported in *E. coli* and later identified in other bacteria, such as *Staphylococcus aureus* [70]. A study of *C. jejuni* confirmed that *clp*B acts in acid resistance and stomach transit [71].

In addition, *dna*K and *gro*EL are the significant heat shock genes, helping bacteria to overcome stressful environmental conditions, such as heat and acid environments [72]. Of these genes, *dna*K, encoding *hsp*70, assists in the protein folding process through their substrate binding and ATPase domains [72, 73], while *gro*EL, encoding *hsp*60, provides a protected cavity in a double heptameric ring structure for the folding of newly synthesized proteins [72].

Another general resistance VF is the elongation factor TU (EF-tu), the most abundant protein in bacterial cells [74]. EF-tu is a GTP-transferase that catalyzes the binding of aminoacyl-tRNA to the ribosome during the elongation stage of cell growth [75]. Current data shows that EF-tu can be inhibited by aminoglycoside antibiotics, which induces mistranslation and bacterial death [76].

### Validation of in silico identified virulence-related genes using PCR assays

Furthermore, we validated the existence of 11 VAT genes, including six virulence (*cad*F, *cia*B, *irg*A, *mvi*N, *pld*A, and *tly*A), two antibiotic resistance [*tet*(O) and *tet*(W)], and three cytolethal distending toxin (*cdt*A, *cdt*B, and *cdt*C of the *cdt* operon) [77] genes, in *A. faecis* and *A. lanthieri* using species-specific PCR-based assays [38]. *A. lanthieri* tested positive for all 11 VAT genes. By contrast, *A. faecis* showed positive for ten genes except for *cdt*B because no PCR assay for this gene was available for this species [38]. However, our comparative genomics analysis identified all three *cdt* (*cdt*A, *cdt*B, and *cdt*C) genes in the reference genomes of *A. faecis* and *A. lanthieri* strains. To validate our detection of the *cdt*B in *A. faecis* AF1078^T^ genome, we aligned the *cdt*B gene of *A. lanthieri* (UnitProt ID: A0A2K9Y5C5) against the protein sequences of *A. faecis* strain AF1078^T^ using BLASTp and identified gene 2,690,353,140 as the *cdt*B gene of *A. faecis* (identity 78%; E-value = 0) (Supplementary Fig. S1). Similarly, *Campylobacter* spp. also showed variable frequency of the *cdt* genes [78].

Of the Cytolethal Distending Toxin encoded by the *cdt* operon [77], *cdt*B is the active subunit, while *cdt*A and *cdt*C work as two regulatory subunits that bind to *cdt*B [77]. The presence of all three genes of the *cdt* operon may indicate that the *A. faecis* strain AF1078^T^ and *A. lanthieri* strain AF1440^T^ could potentially be pathogenic; therefore, further in vitro research is warranted to investigate risk assessment analysis associated with human and animal health. In contrary to these results, studies have shown the absence of the *cdt* genes in *A. butzleri* [15, 79].

In summary, the results of our PCR assays are in congruence with previous studies where a high frequency of *cad*F, *cia*B, *mvi*N, *pld*A, and *tly*A virulence genes was reported in *A. butzleri* and *A. skirrowii* strains [80, 81]. Similarly, *tet*(O) and *tet*(W) antibiotic resistance genes were also detected in both species, which has also been reported in *A. cryaerophilus* [15]. Our findings indicate that tetracycline resistance is prevalent in the genus *Aliarcobacter*.

## Conclusion

This study provided insights into the virulence-related factors identified in the reference genomes of two new *Aliarcobacter* species, *A. faecis* and *A. lanthieri*, using whole genome sequencing, comparative genomics analysis, and qPCR validation. Our results generally showed genes encoding motility and export apparatus, secretory pathways, abiotic stress resistance, and antimicrobial resistance were found in both *A. faecis* and *A. lanthieri.* However, unique genes were also identified for individual species. We acknowledge that further in vitro and in vivo assays are required to evaluate the roles of virulence-related factors in the pathogenicity of *A. faecis* and *A. lanthieri* in human and animal infections.

## Supplementary Information

**Additional file 1: Table S1.** COG gene occurrence in *Aliarcobacter* spp.

**Additional file 2: Figure S1.** The amino acid alignment of the *cdt*B gene of *A. faecis* AF1078^T^ (top) and *A. lanthieri* AF1440^T^ (bottom).

## Data Availability

The genome annotations generated during the current study are available in the JGI IMG/MER repository, https://img.jgi.doe.gov, under analysis ID Ga0136198 (*Aliarcobacter faecis* strain LMG 28519^T^), Ga0136182 (*A. lanthieri* strain LMG 28516^T^), and Ga0225945 (*A. butzleri* strain NCTC 12481^T^).

## Abbreviations

COG: Clusters of Orthologous Group
EF-tu: Elongation Factor TU
Tat: Twin-arginine translocation
NCBI: National Center for Biotechnology Information
QAC: Quaternary Ammonium Compound
PCR: Polymerase Chain Reaction
RND: Resistance Nodulation cell Division
TA: Toxin-antitoxin
UPGMA: Unweighted Pair Group Method with Arithmetic mean
VF: Virulence Factor
VFDB: Virulence Factor Database
VAT: Virulence, Antibiotic resistance and Toxin
