# Supplementary figures and images for: Comparative genomics analysis and virulence-related factors in novel Aliarcobacter faecis and Aliarcobacter lanthieri species identified as potential opportunistic pathogens
Source: BMC Genomics. 2022 Jun 27;23:471. doi: 10.1186/s12864-022-08663-w (PMC9235176; doi:10.1186/s12864-022-08663-w)

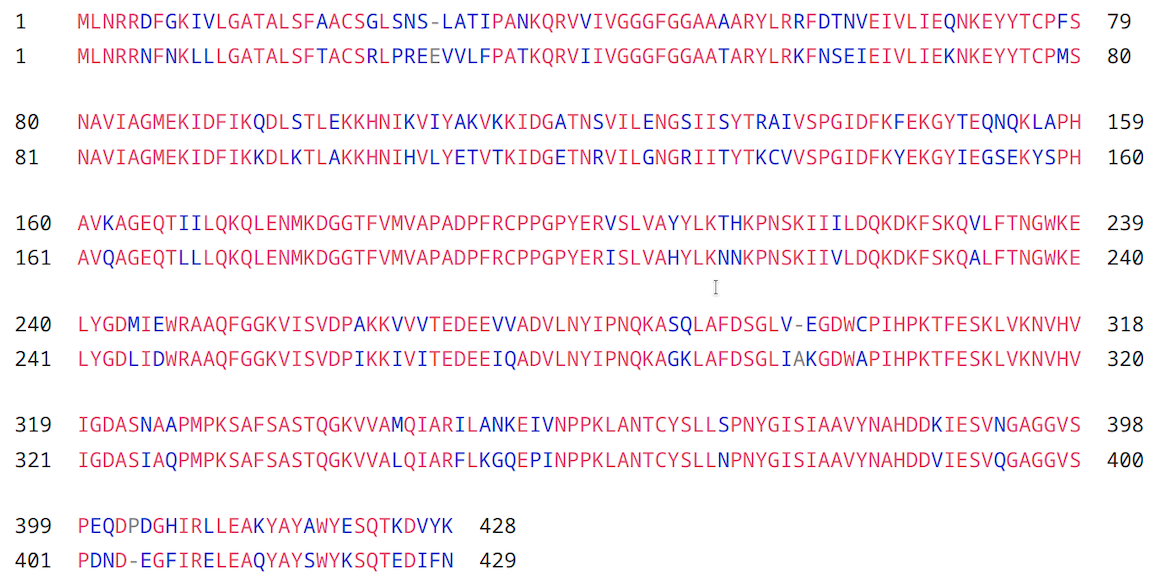

Supplement: Supplementary file 2 — Additional file 2: Figure S1. The amino acid alignment of the cdtB gene of A. faecis AF1078T (top) and A. lanthieri AF1440T (bottom). [file 12864_2022_8663_MOESM2_ESM.tiff]
